# The Ubiquity and Development-Related Abundance Dynamics of *Ophiocordyceps* Fungi in Soft Scale Insects

**DOI:** 10.3390/microorganisms9020404

**Published:** 2021-02-16

**Authors:** Jun Deng, Yuhua Yu, Xu Wang, Qian Liu, Xiaolei Huang

**Affiliations:** State Key Laboratory of Ecological Pest Control for Fujian and Taiwan Crops, College of Plant Protection, Fujian Agriculture and Forestry University, Fuzhou 350002, China; dengjun@fafu.edu.cn (J.D.); yuhuaakx@foxmail.com (Y.Y.); mofazhimeng128@foxmail.com (X.W.); 2200203005@fafu.edu.cn (Q.L.)

**Keywords:** Coccidae-associated *Ophiocordyceps* fungi, obligate fungal symbionts, soft scales, vertical transmission

## Abstract

Mutual relationships with symbionts play a crucial role in the evolution and ecology of plant-feeding hemipteran insects. However, there was no specific dominant bacterium observed in soft scales (Coccidae) in the previous studies, it is still unclear whether soft scales have specific primary symbionts. In this study, a nuclear ribosomal internal transcribed spacer (ITS)gene fragment was used to analyze the diversity of fungal communities in 28 Coccidae species based on next-generation sequencing (NGS). Furthermore, samples from different developmental stages of *Ceroplastes japonicus* were sequenced to illustrate the dynamics of fungal community. Our results showed that Coccidae-associated *Ophiocordyceps* fungi (COF) were prevalent in all 28 tested species with high relative abundance. Meanwhile, the first and second instars of *C. japonicus*, two important stages for growth and development, had high relative abundance of COF, while the relative abundances in other stages were low, ranging from 0.68% to 2.07%. The result of fluorescent in situ hybridization showed that the COF were widely present in hemolymph and vertically transmitted from mother to offspring. Our study confirms that the COF have intimate associations with the growth and development of soft scales, and provides new evidence to support that COF are primary fungal symbionts for Coccidae.

## 1. Introduction

Due to the lack of various essential amino acids in the plant sap, hemipteran insects usually harbor some obligate bacterial symbionts, which provide essential nutrition to complete their diet [1,2]. For examples, *Buchnera* in aphids, *Sulcia* in cicadas, *Tremblaya* in mealybugs, and *Uzinura* in armored scale insects have been reported in many studies [3,4,5,6,7,8]. Normally, these obligate symbionts own extreme genome reduction including several essential amino acid pathways, and provide amino acids and vitamins for insect hosts [6,9]. However, except for the mealybugs and armored scale insects, it has been very ambiguous whether soft scales harbor certain obligate symbionts, which play important roles in growth and development of hosts.

Soft scale insects (Hemiptera: Coccoidea: Coccidae), an economically important plant-feeding group [10], is the third largest family of scale insects, with 1282 species described across 176 genera in the world [11]. Among them, *Ceroplastes rubens*, *Parasaissetia nigra*, *Saissetia coffeae*, *Saissetia oleae*, and *Coccus hesperidum* are well-known polyphagous pests infested on crops and ornamental plants around the world [12]. The newly hatched nymph of soft scales hunts for the appropriate feeding location and becomes immobile after that. Meanwhile, the nymph begins to secrete wax and honeydew, which provides a medium for the growth of some fungi. The small body size and high complexity of morphological identification of soft scales have hindered the progress on the studies of diversity of microbial symbionts for a long time. Flavobacteria (Bacteroidetes) associated with scale insects (superfamily Coccoidea), since 150–250 million years ago, has been considered a potential obligate symbiont in the family Coccidae [9,13]. However, by high-throughput sequencing of bacterial 16S gene, Gomez-Polo et al. (2017) [14] showed that only negligible copy numbers of Flavobacteria were found, and there was no specific dominant bacterium in all seven Coccidae species they sampled. Instead, Gomez-Polo et al. (2017) [14] reported that six healthy soft scale species were dominated by *Ophiocordyceps*-allied fungus (OAF) (Hypocreales, Ophiocordycipitaceae), and vertical transmission of OAF from mother to offspring was observed in two soft scale species using Fluorescence in situ hybridization (FISH), which indicated the possibility of *Ophiocordyceps* being obligate fungal symbionts for soft scales.

Ophiocordycipitaceae was proposed as a new fungal family by Sung et al. (2007) [15]. After that, the species number in this family increased to 358, according to the record of Catalogue of Life [16]. Many fungi of Ophiocordycipitaceae have clavate asci with thickening apices and fusiform ascospores [17]. The dominant hosts of the Ophiocordycipitaceae are arthropods, as well as some nematodes and other fungi [18]. Most Ophiocordycipitaceae species are considered as arthropod or nematode pathogens [18], such as the medicinal fungus *Ophiocordyceps sinensis* [19], the zombie-ant fungus *Ophiocordyceps unilateralis* [20,21], and endoparasitic nematophagous fungus *Hirsutella rhossiliensis* [22]. For scale insects, some *Ophiocordyceps* species have also been identified and observed in the fat body and hemolymph of *Kermes quercus* (Coccoidea, Kermesidae) [23] and *Kerria lacca* (Coccoidea, Tachardiidae) [24], though their function for hosts is not clear. These indicate a potential lifestyle switch from insect pathogens to mutualists might occur for the *Ophiocordyceps* species in soft scales.

Some rare possible mutualistic (or at least nonpathogenic) relationships between *Ophiocordyceps* species and their hosts have been reported in some arthropods, especially in Hemiptera [4]. These studies have investigated an evolutionary process from a prokaryotic symbiosis to a eukaryotic one in certain hemipteran groups. In two species of eared leafhoppers, an endoparasitic *Ophiocordyceps* fungus was observed to replaces obligate symbiont *Sulcia* and Nasuia-allied co-symbiont in an ancestral lineage [25]. The same situation also occurs in Japanese cicada. Matsuura et al. (2018) [4] reported that cicada-parasitizing *Ophiocordyceps* fungi replaced obligate symbiont *Hodgkinia* in 15 species, although the obligate symbiont *Sulcia* still remained in all 24 species. In addition, two types of symbionts (bacterium *Sulcia* and *Ophiocordyceps* symbionts) are simultaneously present in four Deltocephalinae leafhoppers, and form “dual bacterial-fungal symbiosis” [26]. These eukaryotic symbionts might have similar nutritional roles with the obligate bacterial symbionts, which support the growth of hosts feeding solely on nutritionally deficient plant sap. Considering that most *Ophiocordyceps* species are pathogens, this group provides a model system for understanding evolutionary shift from pathogens to possible mutualists associated with their insect hosts.

In the past, based on some short bacterial gene fragments, researchers attempted to reveal symbiont communities in insect species [27,28] as well as determine the evolution relationship between obligate symbionts and their hosts [29,30]. With the development of next generation sequencing (NGS), this technique has been widely used in characterization of symbiont communities in soil, water, and insects [31,32,33]. Based on the results of six tested species that Gomez-Polo et al. (2017) [14] reported, *Ophiocordyceps* fungi might be the primary symbionts in soft scales. However, more tested species were needed to confirm that Coccidae-associated *Ophiocordyceps* fungi (COF) are primary symbionts in Coccidae. Three conditions should be satisfied to determine that a symbiont is a primary symbiont for insect hosts: the first one is that the symbiont is of strict vertical transmission from mother to offspring; the second one is the presence of a symbiont in the majority of species in a single phylogenetic clade; the last one is that the symbiont plays a functional role for insect hosts. To confirm that the COF were primary symbionts in Coccidae, we attempted to demonstrate (1) that the COF should be observed in eggs using the FISH and NGS technique, which indicated vertical transmission; (2) that the COF should be prevalent and dominant in the fungal communities of 28 tested Coccidae species; (3) that the COF should be present in different developmental stages, and had development-related abundance dynamics reflecting functional roles.

## 2. Materials and Methods

### 2.1. Insect Samples

**Figure 1:**
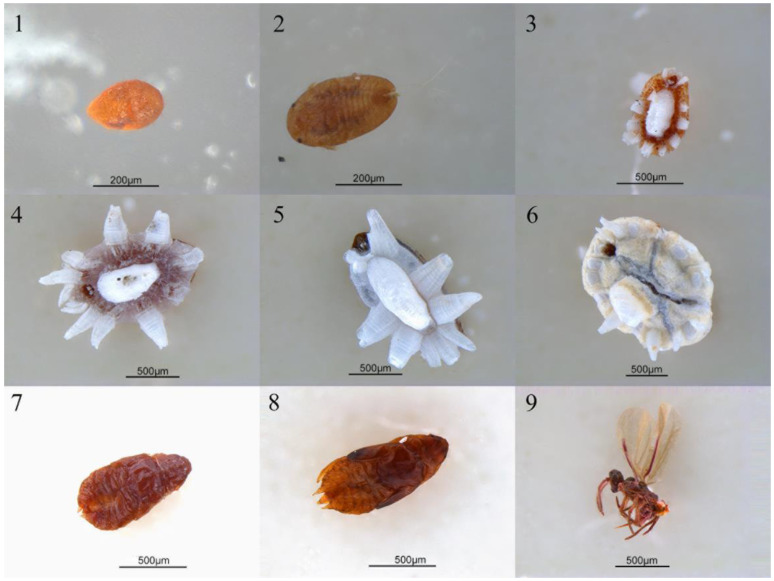
The photos of *Ceroplastes japonicus* at nine developmental stages. 1. Egg; 2. new nymph; 3. first instar nymph; 4. second instar female nymph; 5. second instar male nymph; 6. third instar female nymph; 7. prepupa; 8. pupa; 9. male adult.

**Figure 2:**
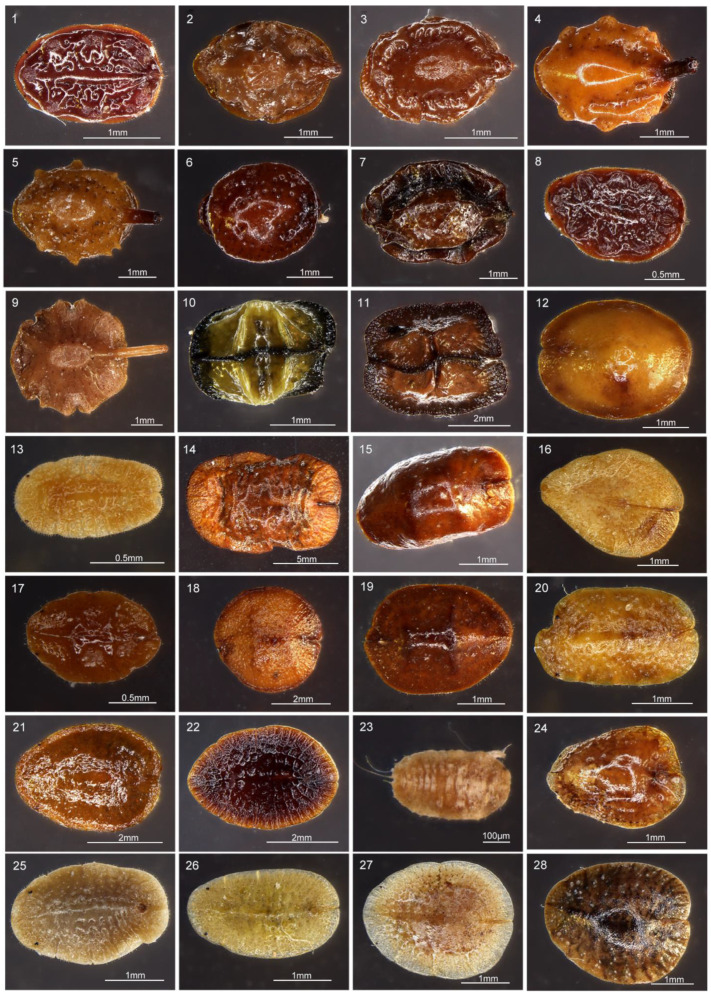
The microphotographs of 28 Coccidae species without wax in this study. 1. Ceroplastes floridensis, 2. Ceroplastes rubens, 3. Ceroplastes cirripediformis, 4. Ceroplastes ceriferus, 5. Ceroplastes pseudoceriferus, 6. Ceroplastes japonicus, 7. Ceroplastes rusci, 8. Ceroplastes kunmingensis, 9. Ceroplastes sp., 10. Dicyphococcus ficicola, 11. Dicyphococcus sp., 12. Ericerus pela, 13. Rhodococcus sariuoni, 14. Megapulvinaria maxima, 15. Saissetia coffeae, 16. Protopulvinaria pyriformis, 17. Saissetia oleae, 18. Saissetia miranda, 19. Saissetia sp., 20. Pulvinaria aurantii, 21. Parasaissetia sp., 22. Eucalymnatus tessellatus, 23. Takahashia japonica, 24. Coccus praetermissus, 25. Pulvinaria psidii, 26. Coccus viridis, 27. Coccus hesperidum, 28. Coccus celatus.

To determine whether the *Ophiocordyceps* were widespread in soft scales, we collected 61 samples from 28 common soft scale species in this study. The samples were field-caught on 28 host plant species from 15 localities in southern China. The information on host plant, locality, and collection date were recorded for each sample (Supplementary Materials Table S1). To show changes of the microbiome profiles associated with *Ophiocordyceps* at different host developmental stages, *Ceroplastes japonicus* was reared on gardenias at 25 °C under long daylight condition (16L:8D) from June to September, 2018, in the laboratory. In total, 25 samples were collected, including the egg, newly hatched nymph (newN), first instar nymph (star1), second instar female nymph (star2F), second instar male nymph (star2M), third instar female nymph (star3F), prepupa, pupa, and male adult (adultM) (Figure 1, Table S1). For male individuals, the stages of prepupa and pupa occurred after the second instar, then became male adult. For female individuals, however, they became female adults after the third instar. The stage of female adult was not included due to insufficient individual numbers. All samples were preserved in 95% ethanol at −80 °C for molecular analysis. All species were identified based on morphological characters [34,35] and molecular identification (Table S1). In the field, soft scales are occasionally parasitized by parasitoid wasps or fungi. Hence, to select healthy individuals for experiments, we observed and filtrated healthy individuals as samples under a stereoscopic microscope (Nikon SMZ18) after removing wax with a dissecting needle (Figure 2).

### 2.2. DNA Extraction, PCR, Cloning, and Sequencing

All individuals without wax were surface-sterilized to avoid contamination from external microbes with 95% alcohol for 1 min, 3% sodium hypochlorite solution for 3 min, and washed three times with distilled water. The DNeasy Blood and Tissue Kit (Qiagen, Dalian, China) was used to extract genomic DNA from whole insect, following the manufacture’s instruction. For most samples, DNA extraction was performed in a pool of 1–5 individuals. For some samples with very tiny body sizes, 10 or more individuals were used. The COI and internal transcribed spacer (ITS) genes of each sample were amplified using specific primers C1-1554F and C1-2342R [36], Hyp2084F and Hyp3489R, CFSZ-CS and LSU-CS-1R [4]; the PCR primers and annealing temperatures are shown in Table S2. PCR products were visualized on agarose gels, and the most intense products were sequenced bidirectionally. The samples with overlapping peaks in sequencing chromatograms were cloned and then sequenced.

### 2.3. Next Generation Sequencing

The symbiotic fungal communities of all samples were assessed by next generation sequencing of the ITS region of small subunit ribosomal RNA gene amplicons, using the Illumina HiSeq platform performed by Biomarker (Beijing, China) Technologies Corporation. The forward primer ITS1F (5’-CTTGGTCATTTAGAGGAAGTAA-3’) and reverse primer ITS2R (5’-GCTGCGTTCTTCATC GATGC-3’) [37] were used to generate amplicon libraries (paired-end, 2 × 250 bp). In order to get high quality data (clean reads), Trimoraic v. 0.33 [38] software was used to filter the raw tags. Forward and reverse sequences were merged into single sequences using FLASH v. 1.2.11 [39] (overlap ≥10 bp and error ration ≤0.2) for each sample. The chimera was identified and removed by UCHIME v. 4.2 [40]. After dereplication and sorting of all sequences, singletons were discarded and the remaining sequences were clustered into representative operational taxonomic units (OTUs) at 97% similarity cutoff using USEARCH (v. 10.0) [41], and 0.05% of all reads was used as a threshold to filter OTUs. The RDP Classifier v. 2.2 [42] was used to annotate OTU species classification based on the taxonomic databases Unite (Release 7.2) [43].

### 2.4. Fungal Diversity in Different Samples

The Chao1 richness estimator, Shannon–Wiener diversity index and Simpson diversity index were calculated in mothur v. 1.30 [44]. The Chao1 richness estimator mainly calculates the minimum number of OTUs in a sample, higher Chao1 value indicates a higher community richness. The Shannon–Wiener diversity index is the average degree of uncertainty of predicting the species of a random sample in a community. The lower the diversity a community has, the lower the Shannon value. The Simpson index is the probability that individuals from two consecutive random samples are the same species. Higher Shannon and lower Simpson values indicate a higher diversity in a community. Finally, principal coordinates analysis (PCoA) was used to visualize difference of the fungal communities at different host developmental stages.

### 2.5. Identification of Unclassified Otus and Phylogenetic Analyses

Some unclassified OTUs were dominant with >30% relative abundance at family level in some samples. We thought these dominant unclassified OTUs might belong to Ophiocordycipitaceae, and their unclassification might be due to having had significant sequence diversity compared to the reference sequences in the taxonomic reference databases. We obtained about 800 bp long ITS sequences representing these OTUs using the Ophiocordycipitaceae primers ITS1F and ITS4R. These ITS sequences were then used to reconstruct phylogenetic trees with known *Ophiocordycipitaceae* species. Forty-five ITS sequences of the Ophiocordycipitaceae were downloaded from GenBank. The phylogenetic analyses were performed to determine the phylogenetic positions of these dominant unclassified OTUs. Phylogenetic trees were reconstructed using Bayesian inference (BI) and maximum likelihood (ML) methods. The program MAFFT v. 7.313 [45] was used to carry out the multiple alignment, and ModelFinder [46] was used to infer the best-fitting evolutionary models via Bayesian information criterion (BIC). The BI analysis was inferred using MrBayes v. 3.2.6 [47] with the GTR + I + G + F model (1,000,000 generations). Meanwhile, ML analysis was performed using IQ-TREE [48,49], with the TPM3u + I + G4 + F model for 5000 ultrafastbootstraps [50]. *Nectria haematococca* and *N. ipomoeae* were selected as outgroups.

### 2.6. Fluorescent in Suit Hybridization

To identity the distribution of *Ophiocordyceps* within a soft scale body, fluorescent in suit hybridization (FISH) was performed on *C. rubens* and *C. japonicus* with specific probe Hyp760 (CY5-5′-CCTGCCTGGAGCACTCT-3′) [4]. Eggs and nymphs were used in this experiment. The samples were placed in 1.5 mL centrifuge tubes. At first, surface disinfection and cleaning were performed with 95% alcohol, then all samples were fixed in Carnoy’s solution (60% ethanol, 30% chloroform, 10% glacial) overnight at room temperature. After removing the Carnoy’s solution, they were rinsed with 100% alcohol, for three times, to ensure no reagent remains. Then samples were immersed in alcoholic 6% H_2_O_2_ solution to quench autofluorescence at room temperature for almost one week; during this time the H_2_O_2_ solution was changed once every two days. When samples were colorless, the solution was discard and samples were rinsed with 100% alcohol for three times. After rinsing the centrifuge tube with 1 mL 37 °C preheated hybridization buffer (20 mM Tris-HCl (pH 8.0), 0.9 M NaCl, 0.01% SDS, 30% formamide), the hybridization solution with probes (final concentration 10 pm/mL) was added under dark conditions, staining for 12–14 h at 37 °C. After the hybridization, the remaining probes were washed off with PBS TX containing 0.3% Triton X-100. At last, slides were made and observed under a Leica TCS SP8X DLS confocal microscope. Insects with no-probe staining were used as negatives controls.

## 3. Results

### 3.1. Symbiotic Fungal Composition in Scale Insects

In total, high-throughput sequencing yielded 3,890,910 paired reads from 61 samples of 28 soft scale species. After merging and quality filtering, 3,608,175 clear tags were selected for further analyses. At least 21,111 clean tags were obtained for each sample, with an average of 59,150 clean tags per sample. The final tags were classified into 475 OTUs representing 6 phyla, 24 classes, 50 orders, and 102 families, with an average of 39 OTUs per sample.

**Figure 3:**
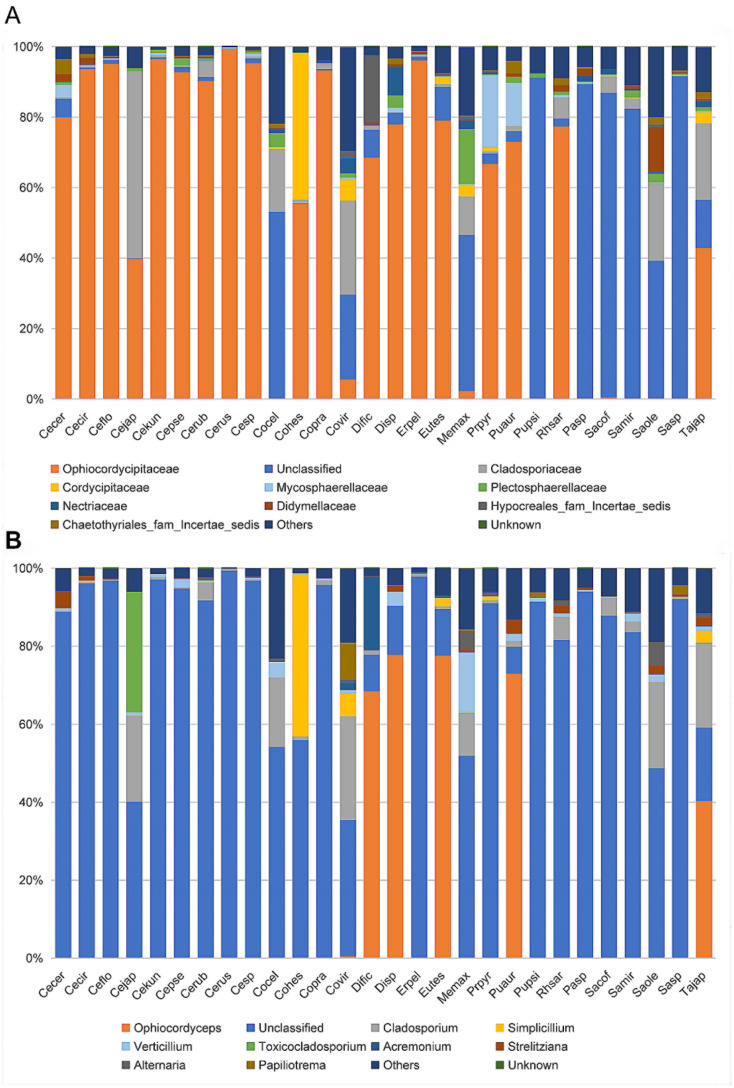
Relative abundance of fungi in 28 Coccidae species at family (**A**) and genus (**B**) levels as assessed by high-throughput amplicon sequencing of the internal transcribed spacer (ITS) gene. *Ceroplastes ceriferus* (Cecer, 1), *Ceroplastes cirripediformis* (Cecir, 3), *Ceroplastes, floridensis* (Ceflo, 4), *Ceroplastes japonicus* (Cejap, 1), *Ceroplastes kunmingensis* (Cekun, 1), *Ceroplastes pseudoceriferus* (Cepse, 3), *Ceroplastes rubens* (Cerub, 4), *Ceroplastes rusci* (Cerus, 1), *Ceroplastes* sp. (Cesp, 3), *Coccus celatus* (Cocel, 1), *Coccus hesperidum* (Cohes, 1), *Coccus praetermissus* (Copra, 1), *Coccus viridis* (Covir, 3), *Dicyphococcus ficicola* (Dific, 3), *Dicyphococcus sp.* (Disp, 1), *Ericerus pela* (Erpel, 3), *Eucalymnatus tessellatus* (Eutes, 3), *Megapulvinaria maxima* (Memax, 1), *protopulvinaria pyriformis* (Prpyr, 3), *Pulvinaria aurantii* (Puaur, 1), *Pulvinaria psidii* (Pupsi, 1), *Rhodococcus sariuoni* (Rhsar, 1), *Parasaissetia* sp. (Pasp, 4), *Saissetia coffeae* (Sacof, 2), *Saissetia miranda* (Samir, 2), *Saissetia oleae* (Saole, 1), *Saissetia* sp. (Sasp, 5), *Takahashia japonica* (Tajap, 3). The number in the bracket represents the sample number of each species.

At the family level, the cluster analysis was performed on the representative OTU. Ophiocordycipitaceae species were detected in all 28 species, and the average relative abundance was 54.36% (standard deviation (SD): 39.54%) (Table S3). The relative abundances of Ophiocordycipitaceae species showed variation among 28 different host species, ranging from <0.1% in *Pulvinaria psidii*, *Saissetia miranda,* and *Coccus celatus* to >95% in *Ceroplastes* sp., *C. floridensis, C. rubens, C. kunmingensis,* and *Ericerus pela* (Table S3; Figure 3A). For eight species (*Megapulvinaria maxima*, *Co. celatus*, *Parasaissetia* sp., *Saissetia* sp., *S. oleae S. coffeae, S. miranda,* and *Pulvinaria psidii*), some unclassified OTUs were dominated with an average of 72.20% (SD: 21.10%) relative abundance, ranging from 39.16% in *S. oleae* to 92.51% in *Saissetia* sp. These unclassified OTUs were confirmed as *Ophiocordyceps* species in subsequent analysis. For *C. japonicus* and *Co. viridis* species, Cladosporiaceae was dominant, accounting for 53.00% and 26.65%, respectively. The relative abundance of *Ophiocordycipitaceae* decreased to 39.89% and 24.04% for *C. japonicus* and *Co. viridis*, respectively. For the remaining 18 species, Ophiocordycipitaceae dominated the fungal communities with averaged relative abundance of 81.87% (±15.42%). At genus level, a large number of OTUs could not be classified, the average relative abundance of unclassified sequences in all sample was 72.80% (±33.47%) (Table S3; Figure 3B). *Ophiocordyceps* was dominate in *Dicyphococcus* sp. (77.79%), *Pulvinaria aurantii* (73.03%), *Eucalymnatus tessellatus* (76.92%), *Takahashia japonica* (41.16%), and *Dicyphococcus ficicola* (69.71%). Some OTUs classified as *Ophiocordycipitaceae* species could not be identified at the genus level, indicating they had noteworthy sequence diversity compared to other *Ophiocordycipitaceae* species.

To evaluate the alpha diversity of microbial composition among 28 different species, we calculated the Chaos richness index, the Simpson, and Shannon diversity estimator (Table S3). We found that the fungal community of *Megapulvinaria maxima* had the highest community richness and diversity (Chaos, 78; Shannon, 2.77; Simpson = 0.12), and *Ceroplastes rusci* had the lowest community richness and diversity (Chaos, 18; Shannon, 0.05; Simpson, 0.99).

### 3.2. Identification of Dominant Unclassified OTUs

**Figure 4:**
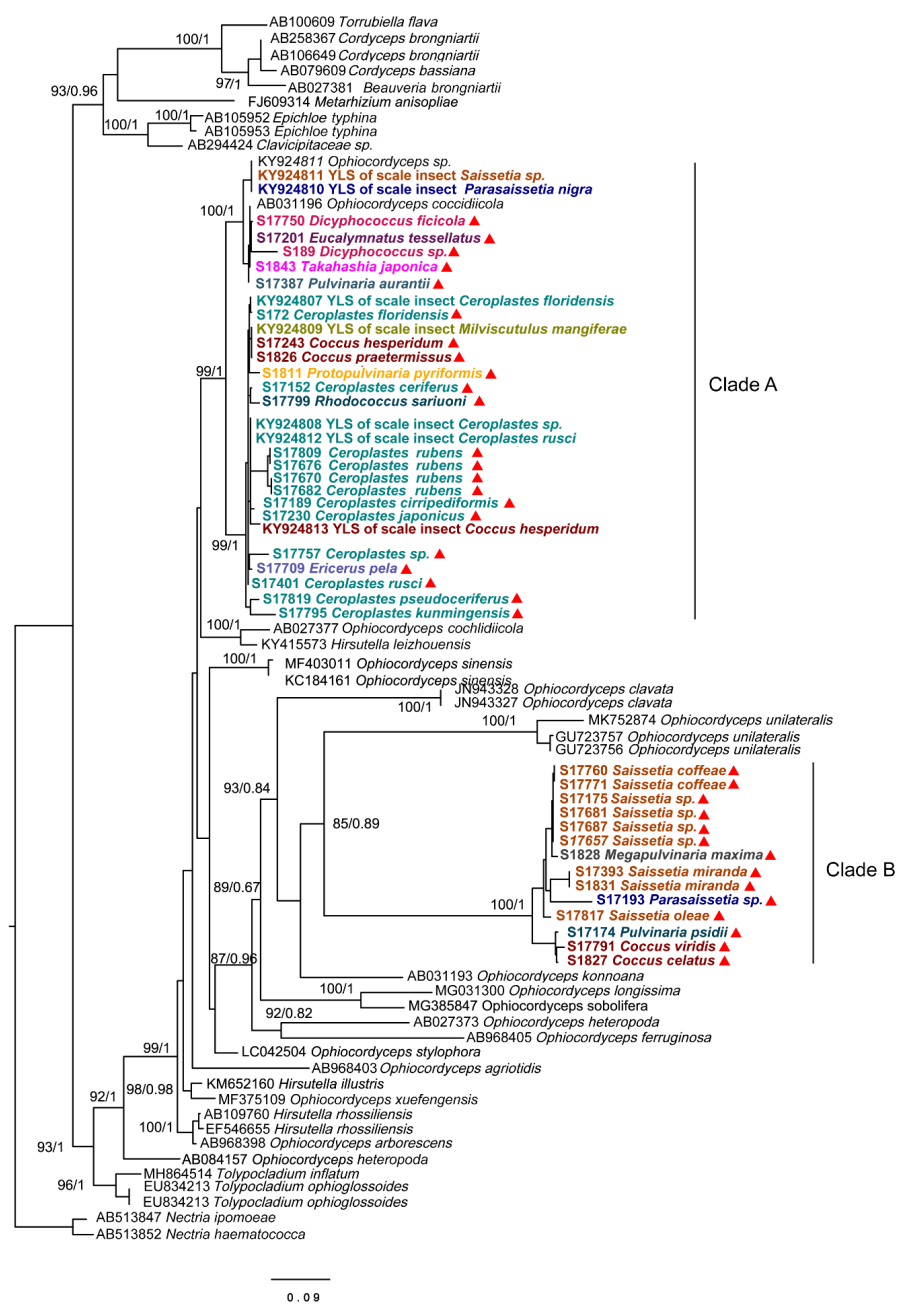
Phylogenetic analysis of the *Ophiocordyceps* fungi based on an (about) 800 bp fragment of partial ITS gene. Bayesian inference (BI) and maximum likelihood (ML) trees had similar topology. The bootstrap support values and posterior probabilities are indicated for each node. Support values below 70 are not shown. The samples in this study are labeled with red triangles. Different colors represent species from different genus.

**Figure 5:**
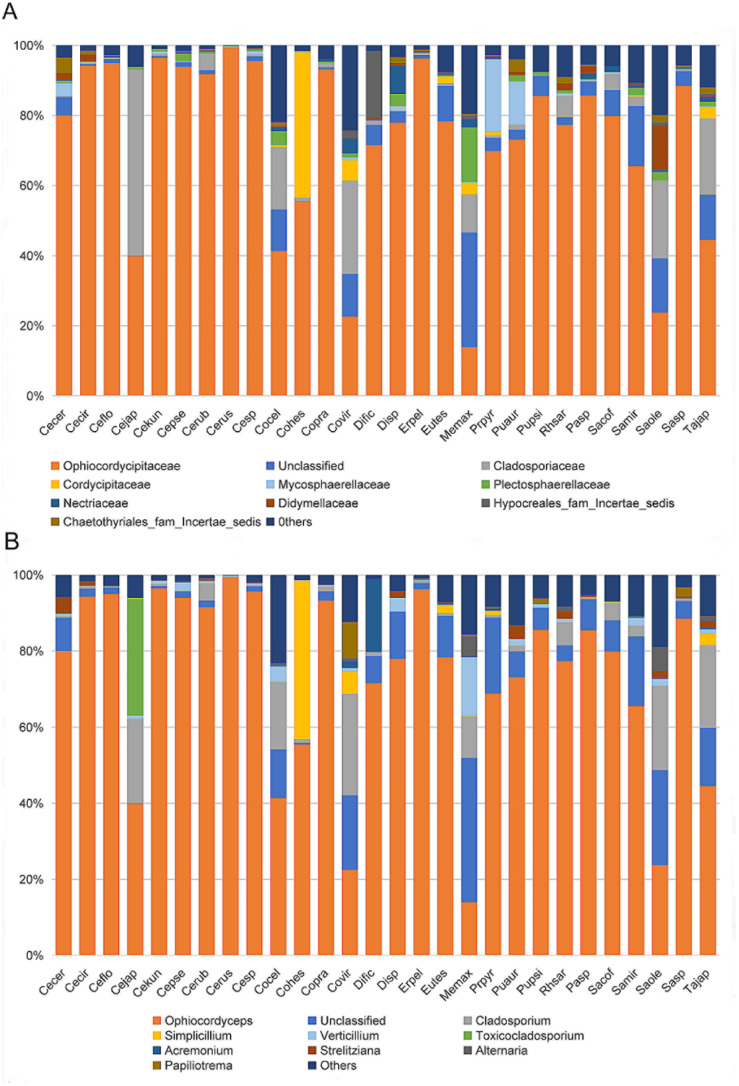
Relative abundance of fungi in 28 Coccidae species at family (**A**) and genus (**B**) levels after some unclassified operational taxonomic units (OTUs) were confirmed as *Ophiocordyceps* species. Species Abbreviations are the same as those in Figure 3.

To identity the dominant unclassified OTUs, we amplified and obtained an (about) 800 bp fragment of the ITS gene from each sample using the Ophiocordycipitaceae primers ITS1F and ITS4R. By aligning these ITS sequences with the dominant OTUs sequences, we finally confirmed these ITS sequences represented those dominant unclassified OTUs. Phylogenetic analysis using ML and BI generated almost congruent tree with relatively high posterior probabilities and bootstrap values (Figure 4). The dominant unclassified OTU sequences were nested inside the *Ophiocordyceps* in the phylogenetic tree, which confirmed the dominant unclassified OTUs belonged to the *Ophiocordyceps* species. The *Ophiocordyceps* sequences from 28 Coccidae species formed two major clades (A and B) in the phylogenetic tree. The clade A included those *Ophiocordyceps* from *Ceroplastes*, *Dicyphococcus, Eucalymnatus, Takahashia, Milviscutulus, Rhodococcus,* and *Ericerus* species. Notably, all *Ophiocordyceps* sequences from seven *Ceroplastes* species were clustered together in the clade A. The Clade A also included a pathogenic fungus of scale insects, *Ophiocordyceps coccidiicola* (AB031196). Correspondingly, all *Ophiocordyceps* sequences from *Saissetia* and *Parasaissetia* species of our samples were included in the clade B. The *Ophiocordyceps unilateralis* was closed to the clade B, which was a pathogenic fungus of ants. The *Ophiocordyceps* sequences of the genus Coccus and Pulvinaria were scattered in the two clades. Additionally, some *Ophiocordyceps* species from *Saissetia* and *Parasaissetia* sampled from *Ficus carica* in Israel and Cyprus in Gomez-Polo et al. (2017) [14] were included in the clade A. After identifying the dominant unclassified OTUs, we replotted the relative abundance of fungi in 28 *Coccidae* species (Figure 5A,B). The mean relative abundance of *Ophiocordycipitaceae* species were 72.55% (SD: 25%), the value was the same at the genus level.

### 3.3. Abundance Dynamics of Ophiocordyceps across Different Developmental Stages of Ceroplastes japonicus

**Figure 6:**
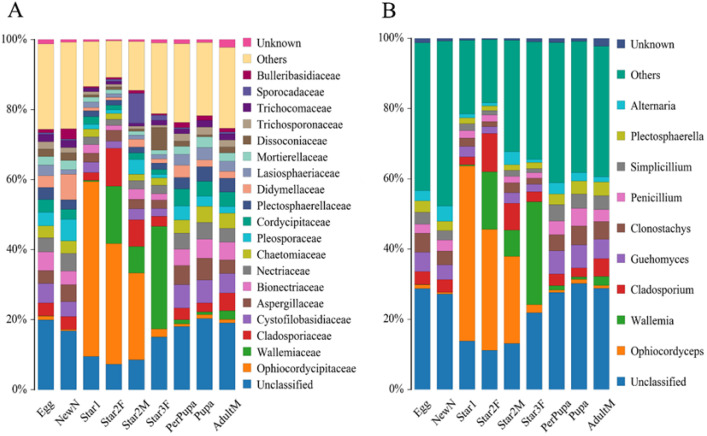
Relative abundance of fungi at family level (**A**) and genus level (**B**) in *Ceroplastes japonicus* at nine developmental stages, including the egg, newly hatched nymph (newN), first instar nymph (star1), second instar female nymph (star2F), second instar male nymph (star2M), third instar female nymph (star3F), prepupa, pupa, and male adult (adultM).

A total of 2,000,620 pairs of reads from the 25 samples were obtained. After merging and quality filtering, 1,784,292 clean tags were generated. Each sample produced at least 68,867 clean tags. High-quality sequences were clustered into 2350 OTUs, corresponded to 4 kingdoms, 16 phyla, 48 classes, 107 orders and 253 family. The average relative abundance of the total unclassified OTUs was 14.9%, the dominant unclassified OTU was from the class Agaricomycetes of phylum Basidiomycota, with mean 5.2% relative abundance. The *Ophiocordyceps* species was directly identified based on the taxonomic reference databases. At the family level, Ophiocordycipitaceae was observed in each sample with varied relative abundance in different developmental stages (Figure 6A, Table 1). The first instar nymph contained the highest relative abundance of Ophiocordycipitaceae, accounting for 49.91%. For the second and third instars, the relative abundance of Ophiocordycipitaceae decreased gradually with the nymph development. The relative abundance decreased to 2.05% in the third instar. The low relative abundances of Ophiocordycipitaceae were observed in stages of prepupa, pupa, and adult male. Interestingly, the second instar female nymphs had much higher relative abundance of Ophiocordycipitaceae (34.49%) than the second instar male nymphs (24.94%). At the genus level, the results were similar to those at family level (Figure 6B). *Ophiocordyceps* predominated in the first and second instar nymphs compared to the relative low relative abundances for other instar nymphs (Table 1). The changes in relative abundances did not mean change in absolute numbers of the *Ophiocordyceps*, and were a good way to show symbiotic fungal composition changes. *Ophiocordyceps* was found in the egg, indicating that vertical transmission occurred from the female parent to offspring.

**Figure 7:**
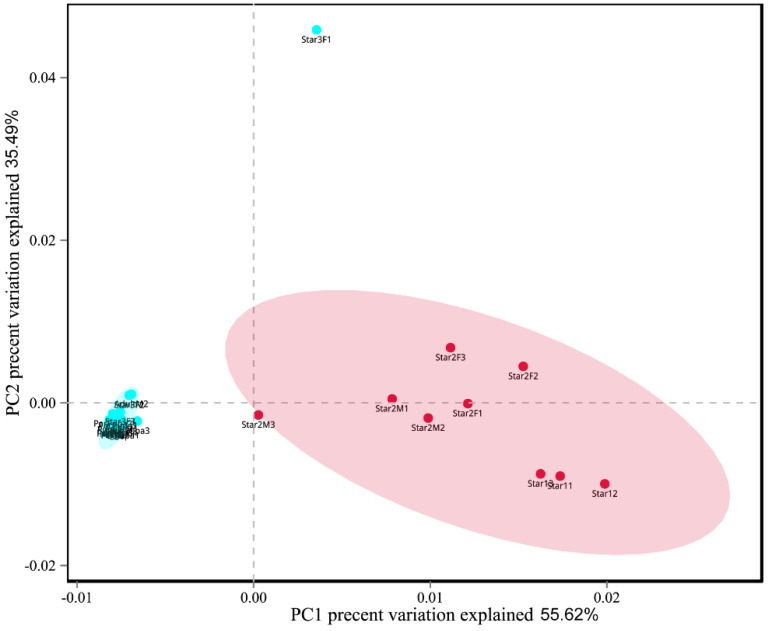
Principal component analysis scatter diagram of microbial community structure of *Ceroplastes japonicus* among nine development stages. Each point represents a sample, different colors indicated different clusters. Abbreviations are the same as those in Figure 6.

The Chaos richness indexes, the Simpson, and Shannon diversity estimators are shown in Table 1. Egg, new nymph, prepupa, pupa, and adult stage had the higher values of Shannon index and lower values of Simpson index than other stages. This result suggested higher community diversity in these developmental stages, and also indicated that *Ophiocordyceps* was dominant in the other life stages (instars 1–2). The Chao index of star3F was highest, and lowest in the pupa. It indicated that the greatest fungal community richness was in the third instar female nymph compared to lowest community richness in the pupa. The dissimilarity of the communities from different samples was visualized using principal component analysis (PCA) (Figure 7). We used the first two PCA dimensions as they explained 91% of the variation. The composition of fungal communities of the adult, pupa, prepupa, egg, and new nymph were similar and clustered together, whilst the fungal communities of the first and second instar nymph were in the other cluster.

### 3.4. Fluorescence in Suit Hybridization

**Figure 8:**
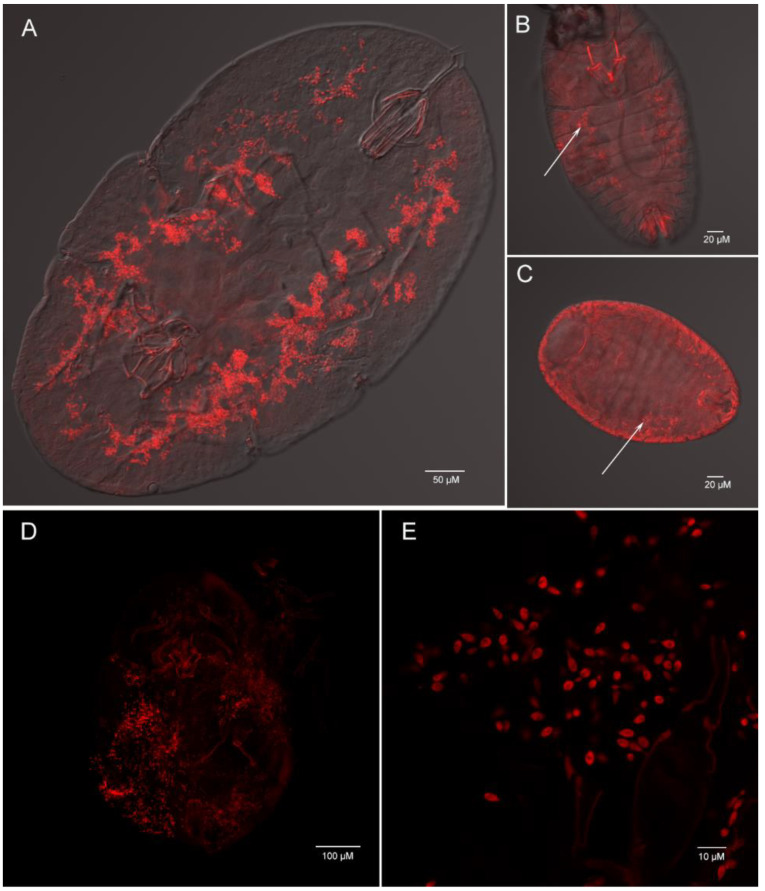
Whole mount fluorescence in situ hybridization of *Ophiocordyceps* fungi (red) in *Ceroplastes rubens* (**A**,**B**,**C**) and *Ceroplastes japonicus* (**D**,**E**). Nymph (**A**), new nymph (**B**) and egg (**C**) of *Ceroplastes rubens*; (**D**) nymph of *Ceroplastes japonicus*; (**E**) high magnification of *Ophiocordyceps* cells in *Ceroplastes japonicus*. The arrows show the location of *Ophiocordyceps* fungi.

Using whole insect fluorescence in suit hybridization (FISH), we determined that *Ophiocordyceps* are both presented in *C. rubens* and *C. japonicus* with the lemon-shape cells (about 6–8 µm long and 1–3 µm wide) (Figure 8A–E). In the nymph, plenty of *Ophiocordyceps* were widely distributed in hemolymph (Figure 8A). In the egg and new nymph, a few *Ophiocordyceps* were also found (Figure 8B,C), proving that *Ophiocordyceps* was vertical transmission from mother to offspring in soft scales.

## 4. Discussion

*Ophiocordyceps* was clearly observed in the egg of *C. japonicus,* based on the methods of FISH and NGS, indicating the vertical transmission of *Ophiocordyceps* occurred from mother to offspring. A similar result has been confirmed in the egg of *Parasaissetia nigra* by Gomez-Polo et al. (2017) [14]. The vertical transmission of yeast-like symbionts (*Ophiocordyceps* species located within specialized cells in the fat body) have been observed in some cicadas [4]. These evidences in different species confirm that COF are vertical transmission. Strict vertical transmission of symbionts is typically (characteristically) observed for the primary symbiont [51]. However, many facultative symbionts are also vertically transmitted [52], for example, all species of the genus Rickettsia are vertically transmitted in invertebrates [53]. More evidence is needed to support the hypothesis that the COF are primary symbionts for soft scales.

Generally, a primary symbiont should be the presence of a symbiont in the majority of species in a single phylogenetic clade. Although the primary symbiont might be lost or replaced by other symbionts in some species of an insect group [9,54], they should still occupy the majority of that clade, indicating stable and ancient symbiosis. Gomez-Polo et al. (2017) [14] have confirmed that COF, widely known as entomopathogenic fungi, were prevalent in six tested species from soft scales. Here, 28 different Coccidae species from 14 localities were collected and analyzed in our study. Our result confirmed that COF were presented in all 28 tested species with high relative abundance, indicating that the symbiosis was stable.

The obligate and vertically transmitted symbionts have reduced genomes or lost some genes considered essential compared to their free-living relatives [52,55], and play functional roles at different developmental stages of their hosts. Genome sequencing of the Coccidae-associated *Ophiocordyceps* fungi are a key way for illustrating their biological and ecological roles in scale insects. However, the specific contribution and role of *Ophiocordyceps* in soft scales are still unknown. In our study, a very important and noteworthy work is to analyze the composition change of the fungal community over different developmental stages of *C. japonicus*. The COF appeared in all developmental stages, especially in the first and second instar, with high relative abundance, showing an intimate association with their hosts’ growth and metabolism. The time during the first and second instar is important for feeding and growth, the length of the body increases from about 300 um at the newly-hatched stage to 700 um at the second instar, close to the size of the female adult. At the second instar male, third instar female, prepupa, pupa, and male adult stage, low relative abundances of COF indicate that the contribution to pupa, mating, and reproduction of COF might not be remarkable. The fungal symbionts have large genome and provide a much more complete metabolic potential and synthesize all protein amino acids relative to bacterial symbionts [56]. Some examples have shown substitutive processes, from a bacterial symbiosis to a fungal one in some hemipteran groups [4,25]. Fungal symbiosis with larger genome revealed its metabolic versatility, including synthesis of almost all amino acids, vitamins, and other metabolites, which is sufficient to compensate for bacterial symbiosis loss [4,56,57]. Based on the above evidences in our study, we finally considered that *Ophiocordyceps* fungi are primary symbionts for soft scales.

In general, obligate symbionts have a long history of codiversification with their hosts, due to mutualism and indispensability for each other [14,54]. Many obligate bacterial associations of insects from 40 to 280 MYA [52]. Interestingly, the oldest evidence of animal parasitism by fungi (*Ophiocordyceps*) is from a fossil of a male scale insect estimated in the Upper Albian of the Lower Cretaceous (99–105 Mya) [18], indicating there is a long history between the *Ophiocordyceps* species and scale insects. In the phylogenetic tree, most hosts of *Ophiocordyceps* fungi were insects, indicating a strong relationship between pathogenic fungi and COF. The *Ophiocordyceps* of *Saissetia* clade is close to the zombie-ant fungus *O. unilateralis* in the phylogenetic tree, indicating that the *Ophiocordyceps* clade associated with the *Saissetia* clade might be related to ants’ pathogenic fungi. Ant attendance of scale insects is a well-known phenomenon [58,59]. Host shifts can be promoted by the overlapping ecological niche of the unrelated hosts [60]. Interestingly, *Ophiocordyceps* of *Saissetia* and *Parasaissetia* species collected from Israel and Cyprus [14] clustered with the *Ceroplastes* clade in our study. The COF in the same insect hosts from different countries have different lineages in the phylogenetic tree, providing evidence to support a hypothesis that soft scales might be occasionally and independently affected by *Ophiocordyceps* pathogens from other insects, then *Ophiocordyceps* species gradually reduced and lost their virulence with a long evolutionary history. Finally, some *Ophiocordyceps* species became obligate fungal symbionts in soft scales, which played functionally equivalent roles.

In conclusion, *Ophiocordyceps* fungi are widespread in soft scales, and show intimate association with the growth and development of soft scales. Our study further confirms that COF should be primary symbionts for soft scales, which might be acquired, newly and independently, from pathogenic relatives in the genus *Ophiocordyceps*. Our analysis may indicate a long-term evolutionary switch from insect pathogens to mutualistic symbionts in different hosts. Further works, including genomic and histological research, are needed to investigate the biological function, transmission mechanism, and evolutionary history of *Ophiocordyceps* in soft scales.

## Figures and Tables

**Table 1:** The fungal alpha diversity and relative abundance of Ophiocordycipitaceae in *Ceroplastes japonicus* at different developmental stages (mean ± SD).

Stage	Relative Abundance of Ophiocordycipitaceae	Relative Abundance of *Ophiocordyceps*	Shannon	Simpson	Chaos
Egg	1.11 ± 0.24%	1.11 ± 0.31%	4.88 ± 0.17	0.02 ± 0.00	376.00 ± 62.56
NewN	0.43%	0.43%	4.95	0.02	416
Star1	49.91 ± 2.47%	49.86 ± 3.02%	3.13 ± 0.06	0.25 ± 0.02	338.70 ± 29.59
Star2F	34.49 ± 3.38%	34.48 ± 4.12%	3.36 ± 0.18	0.14 ± 0.02	530.83 ± 95.67
Star2M	24.94 ± 7.87%	24.94 ± 9.64%	3.60 ± 0.06	0.10 ± 0.01	313.94 ± 22.04
Star3F	2.05 ± 2.29%	2.04 ± 2.81%	4.08 ± 0.96	0.13 ± 0.10	682.74 ± 358.03
PerPupa	0.76 ± 0.61%	0.75 ± 0.72%	4.79 ± 0.04	0.02 ± 0.00	338.00 ± 21.10
Pupa	1.13 ± 1.40%	1.09 ± 1.67%	4.60 ± 0.05	0.02 ± 0.00	268.00 ± 1.04
AdultM	0.95 ± 0.26%	0.90 ± 0.30%	4.84 ± 0.11	0.02 ± 0.00	375.58 ± 49.54

## Data Availability

The sequences reported in this paper have been deposited in the GenBank database www.ncbi.nlm.nih.gov/genbank/ (accessed on 24 January 2021) (accession nos. MW380751-MW380786, MW450918-MW450959, and SAMN171362219-SAMN17136304; BioProject nos. PRJNA687140).

## Supplementary Materials

The following are available online at https://www.mdpi.com/2076-2607/9/2/404/s1, Table S1: collection detail of soft scales used in this study. Table S2: primers used for PCR amplification of genes of fungal symbiont and host insects, Table S3: alpha diversity of 28 soft scales through sequencing analysis of ITS gene amplicons and relative abundance.
